# Succinate Dehydrogenase Deficiency: A Treatable Neurometabolic Disorder

**Published:** 2020

**Authors:** Parvaneh KARIMZADEH, Mohammad KERAMATIPOUR, Arezou KARAMZADE, Elham POURBAKHTYARAN

**Affiliations:** 1 Pediatric Neurology Research Center, Research Institute for Children’s Health, Shahid Beheshti University of Medical Sciences, Tehran, Iran.; 2 Pediatric Neurology Department, Mofid Children’s Hospital, Faculty of Medicine, Shahid Beheshti University of Medical Sciences, Tehran, Iran; 3 Department of Medical Genetics, School of Medicine, Tehran, Iran University of Medical Sciences, Tehran, Iran; 4 PhD candidate, Department of Medical Genetics, School of Medicine, Tehran University of Medical Sciences, Tehran, Iran

**Keywords:** Succinate dehydrogenase deficiency, Mitochondrial disorders, Developmental regression

## Abstract

Succinate dehydrogenase (SDH) deficiency is a rare autosomal recessive neurometabolic disorder that causes brain insult, neurodevelopmental delay, exercise intolerance, and cardiomyopathy. A 25-month-old boy was referred to our neurometabolic center due to developmental regression after injecting the influenza vaccine when he was 10 months old. Magnetic resonance imaging (MRI) showed high signal changes in the brain white matter, and magnetic resonance spectroscopy (MRS) detected a high succinate peak at 2.4 parts per million (ppm). The evaluation of urine organic acids showed a significant elevated succinic acid and whole exome sequencing, confirming SDH. Treatment with a mitochondrial cocktail was initiated, and remarkable improvement was observed. SDH deficiency as a treatable neurometabolic disorder should be considered in any patients with developmental disorders, accompanied by hyperintensity in white matter (as similar to leukodystrophia). Further evaluation is recommended since outcomes depend on early diagnosis and treatment.

## Introduction

The succinate dehydrogenase (SDH) enzyme is part of respiratory chain complex II in the mitochondrion, and is essential for converting succinate to fumarate as part of the Krebs cycle. SDH deficiency is a rare autosomal recessive neurometabolic disorder that causes encephalomyopathy in children (1).

Four nuclear genes encode the four subunits of the SDH enzyme, namely *SDHA *(15 exons), *SDHB *(8 exons), *SDHC *(6 exons), and *SDHD *(4 exons), mapping onto chromosomes 5p15, 1p35-p36.1, 1q21, and 11q23, respectively (2). Only a few cases of SDH deficiency have been reported, all resulting from mutations in the gene encoding the SDHA subunit (3). The first reported mutation in the *SDHA *gene was detected in two siblings presenting with Leigh syndrome and SDH deficiency (4). In a study, 23% of patients with respiratory-chain enzyme defects had partial deﬁciencies of succinate dehydrogenase activity in muscle biopsies. In most cases, this reduction could be detected histochemically in biopsies (5).

This study reported a patient with SDH deficiency and elaborated on his treatment and follow up.

## Case Presentation

**Figure 1:**
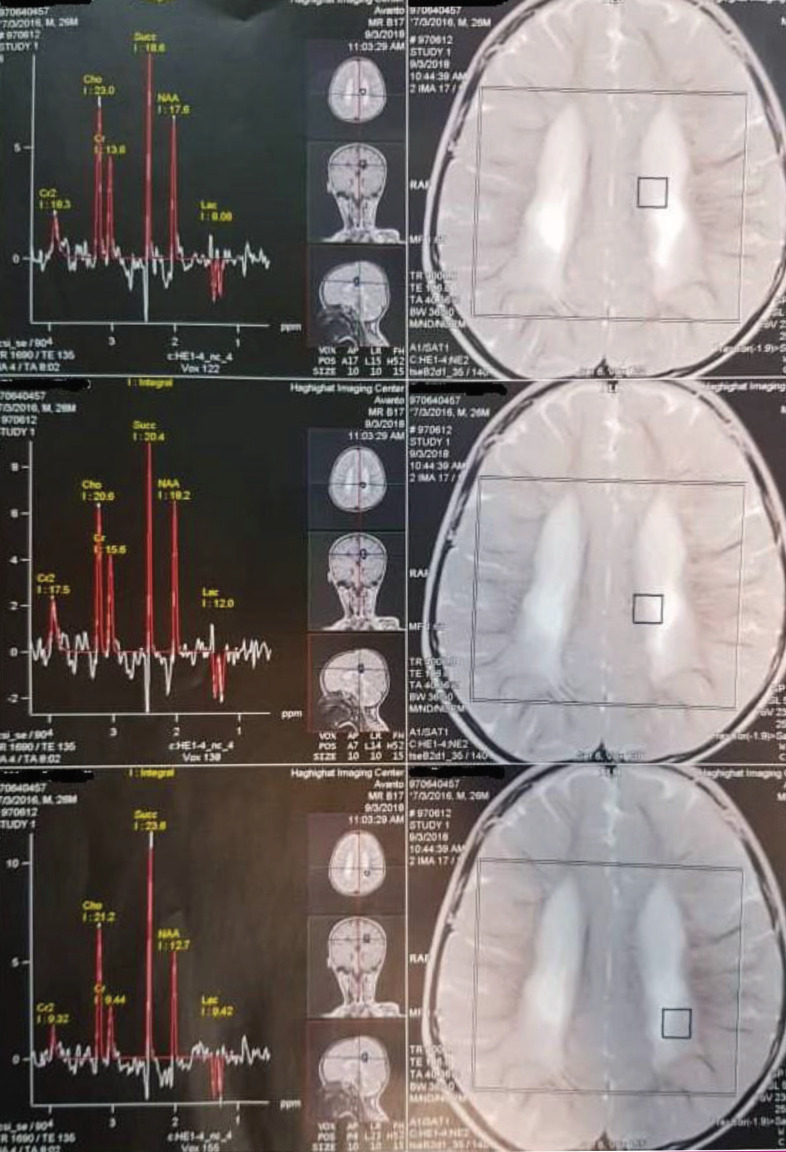
The MRS results showing a remarkable succinate peak

A 25-month-old boy presented with a history of general developmental delay. He was born from relative parents with the gestational age of 32 weeks, birth weight of 1500 grams, and birth head circumference of 32 centimeters. He had no family history of neurological disorders. He was developmentally normal until he was 10 months old and received an influenza vaccine. Gradually, he lost the ability to sit and neck holding. He was unable to say any words and had no history related to the episode of seizure. During the physical examination, he was alert. He could not sit and only had rolling from supine to prone position and vice versa and commando crawling. He had normal deep tendon reflexes (DTRs). He also had normal serum lactate, and the evaluation of his urine organic acids showed an elevated succinic acid (2320 mmol/mol creatinine, with normal range ≤343 mmol/mol creatinine). His MRI results showed a diffused leukodystrophic pattern in the periventricular white matter. Moreover, in his MRS results, a high succinate peak at 2.4 parts per million (ppm) was detected (Figure 1). Therefore, according to our diagnosis compatible with SDH, a mitochondrial cocktail including high doses of riboflavin, co-enzyme Q10, l-carnitine and thiamine, and biotin was initiated. 

In whole-exome sequencing, a homozygous missense variant was detected in the *SDHB *gene. According to the ClinVar database, this variant is classified as a likely pathogenic variant, which causes mitochondrial complex II deficiency. Similarly, this variant can be classified as a likely pathogenic variant based on the American College of Medical Genetics and Genomics (ACMG) guideline (6-8).

**Figure 2:**
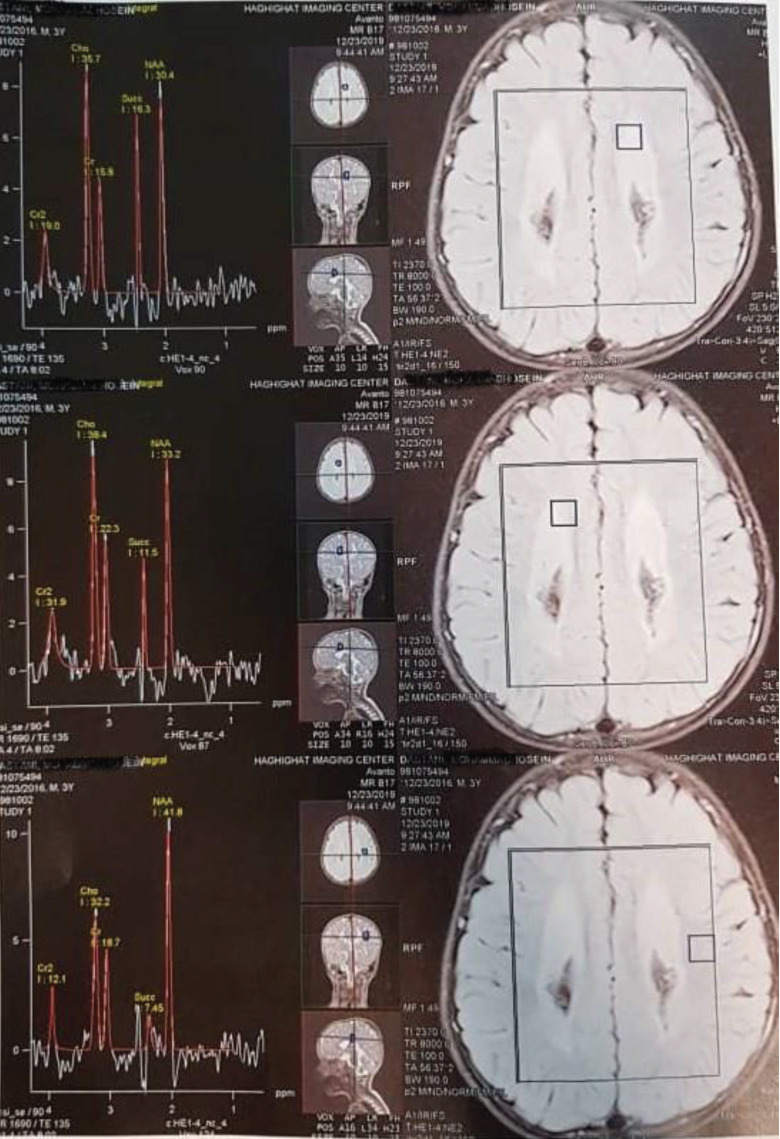
The MRS results after 12 months of treatment showing a remarkable decrease in the succinate peak

After 12 months of treatment, he was able to sit and stand independently for 10 seconds, and was able to express meaningful words. Further, MRS showed remarkable decrease in the succinate peak (Figure 2).

**Genetic analysis:**

At first step of genetic investigations, patient’s blood sample were collected in EDTA containing tubes. Genomic DNA was extracted from whole blood using Blood SV-mini kit (GeneAll Biotechnology Co., LTD, South Korea) according to the manufacturer instruction. Library preparation was performed using Twist Human Core Exome kit (Twist Bioscience, USA) using manufacturer instruction. Sequencing of libraries was done by high-throughput paired-end sequencing using NovaSeq sequencing platform (Illumina Inc., CA, USA). 

Total number of reads obtained for this sample was 57,851,802. These reads generated total sequence of 8,067,368,170 bases that gave an average throughput depth of 244X for target regions. 98.9 percent of target regions had a coverage of more than 1X, and 98.5 percent showed a coverage of more than 10X. Variant calling and filtering was done using Genome Analysis toolkit (GATK-v3.4.0) and detected variants were annotated. Proper filtering and then interpretation of a short list of variants in terms of pathogenicity was performed based on ACMG (American College of Medical Genetics and Genomics) guideline for variant interpretation (6). 

Above investigation resulted in detection of a missense variant (c.143A>T; p.D48V) in exon 2 of SDHB gene (NM_003000.2). This variant was called 54 times out of total depth of 54 at this nucleotide position, showing a homozygous status. 

The detected homozygous missense variant in *SDHB* gene, was reported in Human Gene Mutation Database (HGMD) as a pathogenic variant in patients with complex II deficiency and succinate dehydrogenase-related infantile Leukoencephalopathy (7-8). In addition, *ClinVar *database has classified it as a likely pathogenic variant in its latest clinical evaluation (2018) (9). Multiple lines of *in silico* computational analysis (Mutation Taster, CADD, etc.) support the deleterious effect of this variant on the gene or gene product(s). The variant has very low frequency in population databases (ExAC, 1000G, and our local database). Patient’s phenotype was also consistent with *SDHB*-associated phenotypes. Having such information and based on ACMG guideline, this variant was classified as a pathogenic variant and diagnosis of patient’s phenotype was confirmed. 

## Discussion

Inherited SDH deficiency is a rare autosomal recessive neurometabolic disorder that causes brain involvement, cardiomyopathy, and/or exercise intolerance(1). Few studies have been conducted on SDH deficiency. This study reported a patient with a clinical course and neurological imaging compatible with succinate dehydrogenase deficiency, as confirmed by a genetic study. 

In the patient, developmental milestones were in normal limits but gradually regressed. Furthermore, during physical examination, he was alert and his eyes’ ability to fix and follow was in normal limits. He had regression mostly in motor development, and cognition was less involved. His MRI results showed that signal changes were similar to leukodystrophia. However, he was reported to be alert and seizure free with normal evaluations related to leukodystrophia, and thus, the reliability of this diagnosis was questioned. As a result, we performed MRS, indicating a high metabolite peak in 2.4 ppm, which was assumed to be a succinate peak. After diagnosis, treatment was initiated with riboflavin, coenzyme Q10, and l-carnitine. Moreover, a genetic study was performed for confirmation of the diagnosis, and as we expected, the result was in favor of SDH. Occupational therapy was useful for reducing the spasm. After treatment, the patient improved in different aspects of developmental milestones, including motor skills that initially improved, followed by speech and cognition, respectively.

## In Conclusion

SDH deficiency is a treatable neurometabolic disorder, and its outcomes depend on early diagnosis and treatment. Therefore, it should be considered in any patients with developmental disorders accompanied by hyperintensity in the white matter (as similar to leukodystrophia).
